# Recent progress in the design, synthesis and applications of chiral metal-organic frameworks

**DOI:** 10.3389/fchem.2022.1014248

**Published:** 2022-10-05

**Authors:** Amna Altaf, Sadia Hassan, Bobby Pejcic, Nadeem Baig, Zakir Hussain, Manzar Sohail

**Affiliations:** ^1^ Department of Chemistry, School of Natural Sciences, National University of Sciences and Technology, Islamabad, Pakistan; ^2^ Department of Biomedical Engineering and Sciences, School of Mechanical and Manufacturing Engineering, National University of Sciences and Technology, Islamabad, Pakistan; ^3^ CSIRO Mineral Resources, Australian Resources Research Centre, Kensington, CA, Australia; ^4^ Interdisciplinary Research Center for Membranes and Water Security, King Fahd University of Petroleum and Minerals, Dhahran, Saudi Arabia; ^5^ Department of Materials Engineering, School of Chemical and Materials Engineering, National University of Sciences and Technology, Islamabad, Pakistan

**Keywords:** porous materials, Chiral MOFs, 2D MOFs, isoreticular synthesis, enantioselective membranes, Chiral MOFs in sensing

## Abstract

Chiral Metal-Organic Frameworks (CMOFs) are unique crystalline and porous class of materials which is composed of organic linkers and metal ions. CMOFs surpass traditional organic and inorganic porous materials because of their tunable shape, size, functional diversity, and selectivity. Specific applications of CMOFs may be exploited by introducing desired functional groups. CMOFs have chiral recognition abilities, making them unique for chiral compound synthesis and separation. The CMOFs can be synthesized through different approaches. Two main approaches have been discussed, i.e., direct and indirect synthesis. Synthetic strategies play an essential role in getting desired properties in MOFs. CMOFs find potential applications in adsorption, asymmetric catalysis, luminescence, degradation, and enantioselective separation. The MOFs’ porosity, stability, and reusability make them an attractive material for these applications. The plethora of applications of CMOFs have motivated chemists to synthesize novel MOFs and number of MOFs have been ever-escalating. Herein, the synthetic methods of CMOFs and their various applications have been discussed.

## 1 Introduction

Metal-organic frameworks (MOFs) are porous coordination networks designed by employing various organic linkers and metal ions. MOFs have created a broad class of crystalline materials with exceptional surface area, diverse functionality, versatile composition and higher porosity (Gangu et al., 2016; Gangu and Jonnalagadda, 2021; Rubab et al., 2022). One of other feature of MOFs is that their structure can be rationally modified for some particular applications (Zhao et al., 2021). Since the introduction of these materials in late 1990s, a new area of study has been opened up due to their ability to tailor the pore structure and shape in view of a particular property, enabling the manufacture of rationally designed MOFs (Karmakar et al., 2016). Chirality plays a vital role in explaining life origin, as all the chiral amino acids in enzymes are present in “*L”* form only. The synthesis of chiral compounds is significant for agricultural, pharmaceutics, and food biotechnological industries (Tanase-Grecea et al., 2020). So, chirality and porosity are the crucial features for the materials in different industries. Chiral and porous materials, for example, MOFs and chiral inorganic zeolites, are highly desired due to their potential applications in catalysis, enantioselective separations (Wang et al., 2015), optical devices, chiral separations, medicine**,** asymmetric catalysis, and chiroptical switching (Wen et al., 2018). In recent years, chiral MOFs (CMOFs), particularly CMOFs having entangled systems, exhibited exceptional significance owing to their unique properties (Zhou et al., 2017). The MOFs’ catalytic activity and characteristics can be controlled by tuning its structure with structural components such as functional groups, metal nodes and organic linkers (Berijani et al., 2019).

**FIGURE 1:**
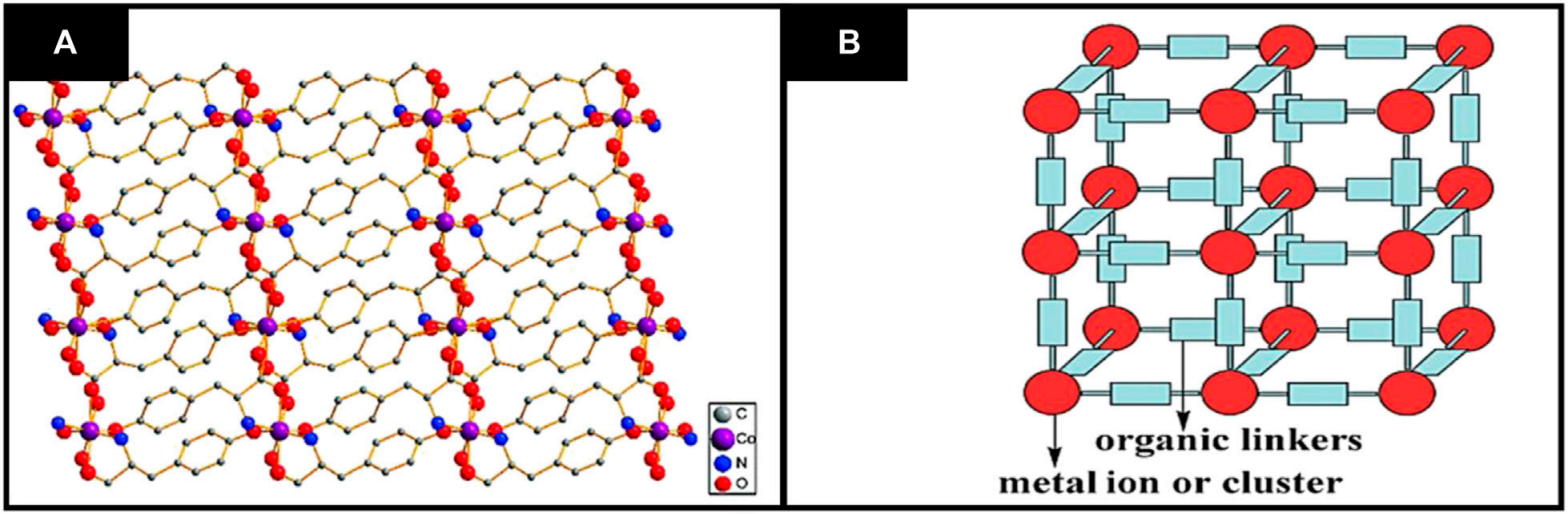
General structure of MOFs **(A)** Two dimensional structural representation of MOFs **(B)** three dimensional structure of MOFs. Reproduced with permission from (Dhakshinamoorthy et al., 2011b).

For instance, various functionalities on organic linkers have played a crucial role in adsorption behavior of MOFs. Different types of organic linkers have been employed during the past few years to synthesize MOFs. In literature, the MOFs are generally developed from stiff linkers as they are stable, and their pore size and shape can be conveniently controlled. MOFs synthesized by using flexible linkers are not much studied (Chen et al., 2011). MOFs’ crystal structure may be predicted based on prior knowledge of their geometry, metal clusters, dimensions preferred and direction of organic linkers (Dhakshinamoorthy et al., 2011a). The general structure of MOFs is shown in Figure 1. Concerning dimensionality, 2D and 3D CMOFs can be synthesized, but 2D nanosheets of CMOFs are highly desired for enhancing their performance in various applications by taking advantage of improved interaction with the guest substrates and better mass transport (Guo et al., 2018).

Active sites in MOFs are incorporated by using three main approaches:

(1) by directly introducing Metallo-ligands for construction of MOF
(2) by loading active species in cavities of MOFs through post-synthetic modification
(3) by using unsaturated coordinative sites of secondary building units (Li et al., 2018). Furthermore, CMOFs can be prepared without using any chiral auxiliaries by the achiral precursors with spontaneous resolution reaction. Conglomerates can be formed by the spontaneous resolution method (Tao et al., 2019).

The first homo-chiral MOF was synthesized in 1999, since then, this field has been developed rapidly. Up till now, more than thirty varieties of CMOFs have been constructed and used to investigate enantioselective adsorption (Gu et al., 2015). It is a great challenge in MOF synthesis to tune chemical properties and chiral environment to control reactivity in a reaction. Another major challenge for chiral MOFs is their less stability to harsh and humid reaction conditions. These issues limit their use in practical catalytic processes (Chen et al., 2017).

As discussed, nature is full of chirality, and fabrications of the CMOFs have a great significance in chiral catalysis, separation and chiral recognition (Han et al., 2018). Due to their wide range of applications, the synthesis of chiral MOFs has become the hot area of research. This review explicitly discusses the synthetic methods and applications of CMOFs in separation, degradation, asymmetric catalysis, capturing, luminescent and adsorbent materials.

Due to multiple applications and synthetic methods, realm of chiral MOFs has garnered attention of scientists and researchers throughout the globe. In literature, multiple studies could be found which had targeted the synthetic methods, separation methods, applications and microscopic structural properties of the chiral MOFs; however, all the information is not concise and succinate rather it is disseminated in different studies. In the first of this study, synthesis processes, their advantages, disadvantages and reported data in the literature are described and then, in second part of study, applications of chiral MOFs are explained. This review is different from previous studies reported in literature on the basis of framework of the paper as it provides a comprehensive overview of both synthesis processes and applications. As this paper is providing the explanation of state of art of chiral MOFs along with their applications, it will provide an in-depth knowledge and information in the field of chirality.

## 2 Synthesis methods of Chiral MOFs

The synthesis of chiral compounds has become an important area of research due to their critical role in chemistry, biology, biotechnology, agriculture and medicine (Morris and Bu, 2010). Two different aspects should be considered while discussing the chirality in the solids; building blocks of the solids are chiral themselves or the solid components arranged in such a way to produce a chiral solid. MOFs are crystalline materials. However, crystallization of organic ligands bonded with the metal ions into the chiral space group is very difficult because of the association of all constituents into the desired symmetries of the chiral space group. There is a myriad of strategies for the synthesis of chiral MOFs; nevertheless, in this paper, these strategies are divided into two broader classes.

• Direct synthesis
• Indirect synthesis

**FIGURE 2:**
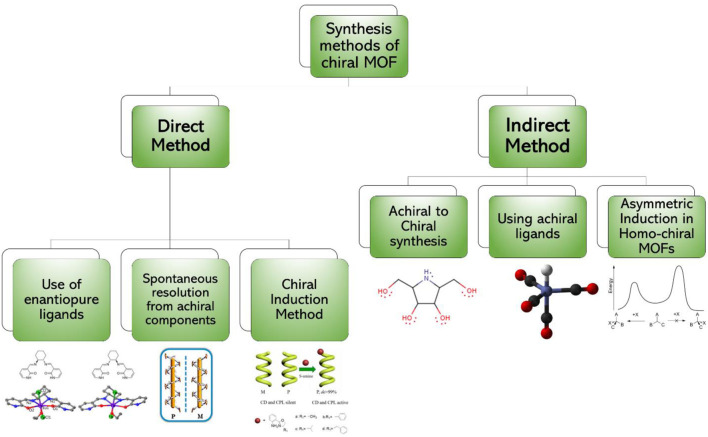
Synthetic strategies of chiral MOFs which are divided into two major categories and each category has further methods for chiral MOF synthesis.

The direct method of synthesis of CMOFs is simpler as compared to other methods; however, the reagents and chemicals are expensive. In addition, their crystallization with other metal ions particularly in low-symmetry chiral space groups is complicated (Ezuhara et al., 1999a). Schematic diagram of synthetic strategies of CMOFs is shown in Figure 2.

### 2.1 Direct synthesis

The direct method is an extensively used method to produce chiral MOFs. In the direct synthesis, chiral reagents are used to achieve CMOFs; the chiral reagents can be chiral linkers attached to the metal cluster or nodes (Gedrich et al., 2011). In some cases, the chiral species is introduced into the MOFs, to produce CMOFs. There are different methods which have been utilized to synthesize CMOFs directly. Details of a few processes and methods are given below.

#### 2.1.1 Use of enantiopure ligands

The enantiopure ligands are available in one specific enantiomeric form. The selection and then synthesis of chiral ligands is important for the construction of chiral MOFs. These ligands are categorized into two different classes.

• Central chiral ligands
• Axial Chiral ligands

**FIGURE 3:**
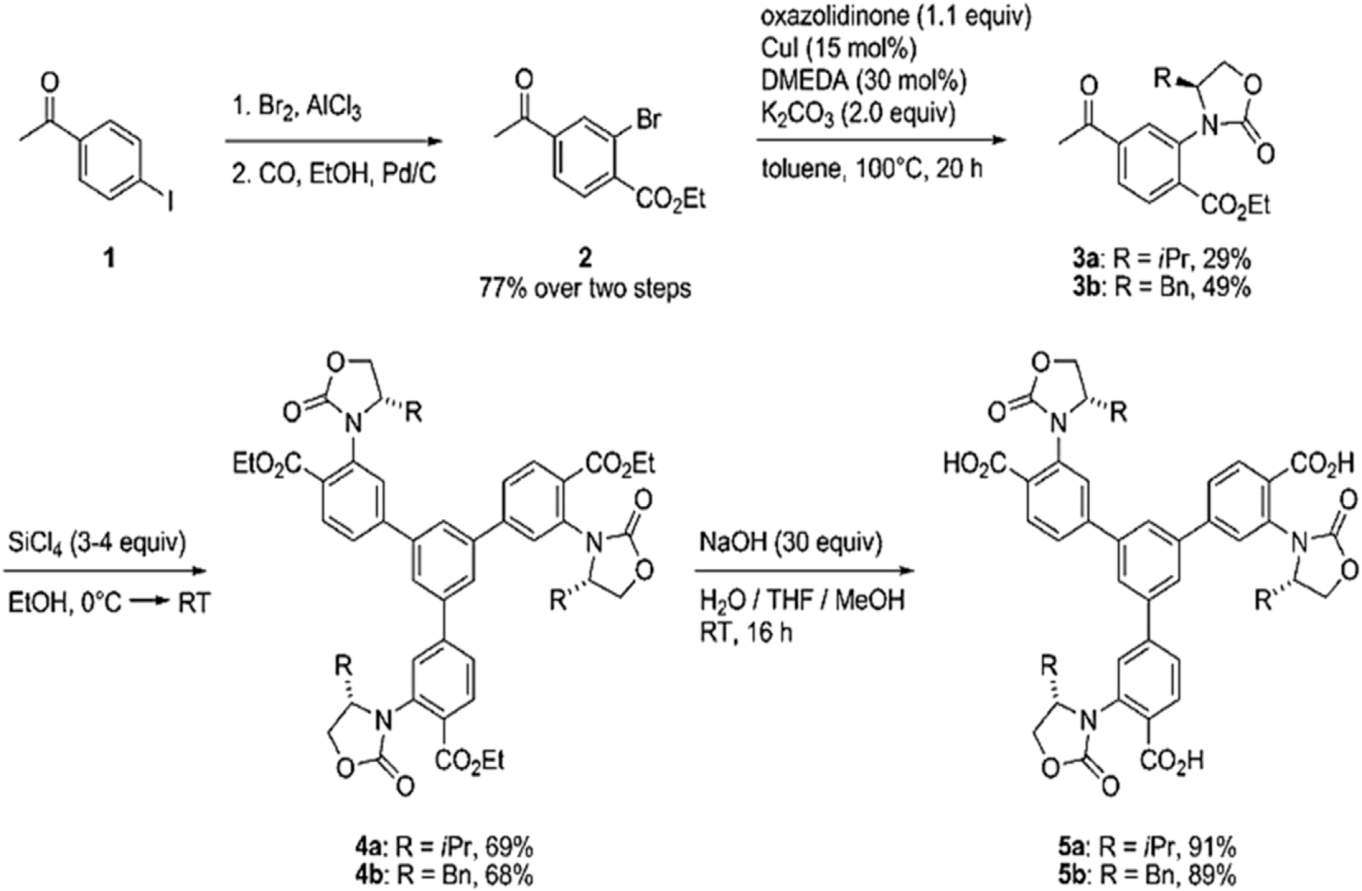
Chiral H_3_BTB-derivatives (5a and 5b) synthesis. Reproduced with permission from (Tao et al., 2019).

These ligands have been used in many studies. For instance, Chen and his co-workers have synthesized the chiral MOFs by incorporating chiral phosphoric acids into indium-based MOFs and preventing them sterically from undergoing coordination. Three types of chiral and 3-D MOFs have been synthesized, which have different topologies, and these MOFs were prepared from 3 enantiopure 1,1′-biphenol-phosphoric acids derived from tetra-carboxylate linkers. The important thing is that the uncoordinated phosphoric acid is aligned periodically in MOFs channels, and it displays higher acidity than non-immobilized acid (Chen et al., 2019). As discussed, the chiral linkers are generally used to produce the CMOFs. Gedrich and his co-workers in 2011 synthesized the CMOFs using multinuclear zinc clusters and trifunctional chiral linkers. Chiral tricarboxylic acids were prepared using BTB as backbone, which is replaced by chiral oxazolidinones (H_3_ChirBTB-n): **5a**, H_3_ChirBTB-1 and **5b**, H_3_ChirBTB-2. Oxazolidinones have been used because these moieties act as chiral auxiliaries in stereoselective synthesis; this BTB ligand is best suited due to its extended and rigid nature. So, the resulting chiral MOF crystals of [Zn_3_(ChirBTB-1)_2_] were synthesized by reacting H_3_ChirBTB-1 (5a) and excess of zinc nitrate in solvent diethyl-formamide at 100°C for 20 h. Using similar reaction conditions but replacing 5a linker with 5b, they synthesized second MOF [Zn_3_(ChirBTB2)_2_]. The synthesis scheme is shown in Figure 3 (Gedrich et al., 2011).

Similarly, Grancha and his co-workers in 2013 synthesized iso-reticular MOFs, which consist of Metallo-ligands. By using this method charge density of MOFs can easily be tuned. In this study iso-reticular, MOFs were synthesized by using chiral oxamidato ligands. The synthesized bio MOFs were novel and had the general formula [Ca^II^Cu^II^_6_L_3_(OH)_2_(H_2_O)].nH_2_O (Bisht and Suresh, 2013; Grancha et al., 2017).

In another study, Jeong et al. reported Cu(NO_3_)_2_.3H_2_O and (S)-1H_2_ reaction in the solvent mixture of DEF/MeOH, placed in N, Nʹ-dimethylaniline to synthesize a chiral MOF [Cu_2_((S)-1)_2_(H_2_O)_2_] ((S)-KUMOF-1). The synthesized MOF has an ample void space, and its organic linker maintains chirality and has functional groups that provide a catalytic site for different reactions (Jeong et al., 2011).

Generally, direct synthesis of chiral MOFs by using enantiopure ligands is very challenging. So, new chiral ligands should be synthesized for construction of new chiral MOFs having nano space for the desired application.

#### 2.1.2 Spontaneous resolution of Chiral MOFs from achiral components

The chiral MOFs can be prepared through spontaneous resolution of achiral components during their crystal growth period. These achiral species form helical structures and entangle all the molecular chains. The homochiral MOF Cd (L_3_)(NO_3_)_2_(H_2_O)(CH_3_CH_2_OH) was reported by Aoyama et al., in 1999 which was based upon achiral 5-(9-anthracenyl)pyrimidine (L3) (Ezuhara et al., 1999a). Many studies reported this method for the synthesis of chiral MOFs (Gil-Hernández et al., 2010; Bisht and Suresh, 2013).

In 2014, Zhang et al. synthesized a helical chiral MOF, [Cu (succinate)(4, 4′-bipyridine)].4H_2_O by reacting 4, 4′-bipyridine and succinic acid with Cu^+2^ ions. The mechanism proposed by them for formation of chiral product was compared with coin flip. When a coin is flipped the probability of negative and positive is equal. Although, when flipped number is less, then situation will be different because the probability of one side will be more than the other (Zhang et al., 2014a). Similarly, for CMOF formation, coordination polymer nucleates, and it causes induction effect for the other, so the complete system will be inclined to one side. The chirality of MOF will be determined from optical property of final product. This process explained that why chiral products are formed rather than racemic mixture by using achiral ligands for helical chain formation (Wu et al., 2007).

Tian and his coworkers in 2005 synthesized a chiral MOF in which ligands act as chiral inductions and plays an important role in helical chain formation (Tian et al., 2005).

Chiral MOF synthesis by using direct method is facile; however, the main challenge is that chiral reagents are very expensive and their crystallization with metal ions is complicated than achiral, and their product is also not enantiopure. So, for synthesis of CMOFs factors affecting crystallization should be studied.

#### 2.1.3 Chiral induction method

CMOFs can also be synthesized by the Chiral Induction method; Zhang and his co-workers in 2015, used this strategy and synthesized chiral MOF-5. This MOF was being synthesized by using H_2_BDC, Zn(NO_3_)_2_·6H_2_O, D-proline or L-proline mixture in water and N-methyl-2-pyrrolidone (Zhang et al., 2015).

Another practical method for chiral MOFs synthesis is chiral induction. First, rather than adhering to MOFs with certain functional groups, the selection of an appropriate inducer for chiral induction can be made for known MOFs. Second, this approach practically never modifies the MOFs skeletons. Furthermore, the characteristics and functions of the emerging chiral MOFs can be regulated by already synthesized MOFs. The main flaw of this strategy is that choosing a template is not universally applicable, which makes it extremely difficult to use in CMOFs synthesis.

### 2.2 Indirect synthesis

For the decades, scientists have been trying to investigate and bring better and efficient methods of CMOFs synthesis. Direct synthesis of chiral MOFs has its limitations including availability of limited recognition sites (Kou et al., 2018) and ability to jeopardize the chiral integrity during reaction (Hu et al., 2016). Therefore, there are several indirect methods of synthesis of chiral in which achiral components and sometimes chirality induction methods are used (Bisht et al., 2015). In this method, the phenomenon of symmetry breaking used. List of chiral and achiral ligands is shown in Table 1. There are many methods to indirectly synthesize CMOFs. A few of such methods are explained below.

**TABLE 1:** List of some chiral and achiral ligands.

Chiral ligands	Achiral ligands
Substituted 1,3-dioxolane-4-carboxylic acid (Han et al., 2018)	L_3_ (Ezuhara et al., 1999b)
Camphor Acid (Gu et al., 2016)	Imidazole (Zhang et al., 2017b)
Hydroxy acid (Han et al., 2018)	Ethane diamine (Dong et al., 2014b)
Dipeptide carnosine (Katsoulidis et al., 2014)	—
L_4_ (Han et al., 2018)	—
L_5_ (Han et al., 2018)	—
1,2-pd (Kepert et al., 2000)	—

#### 2.2.1. Achiral to Chiral transformation

**FIGURE 4:**
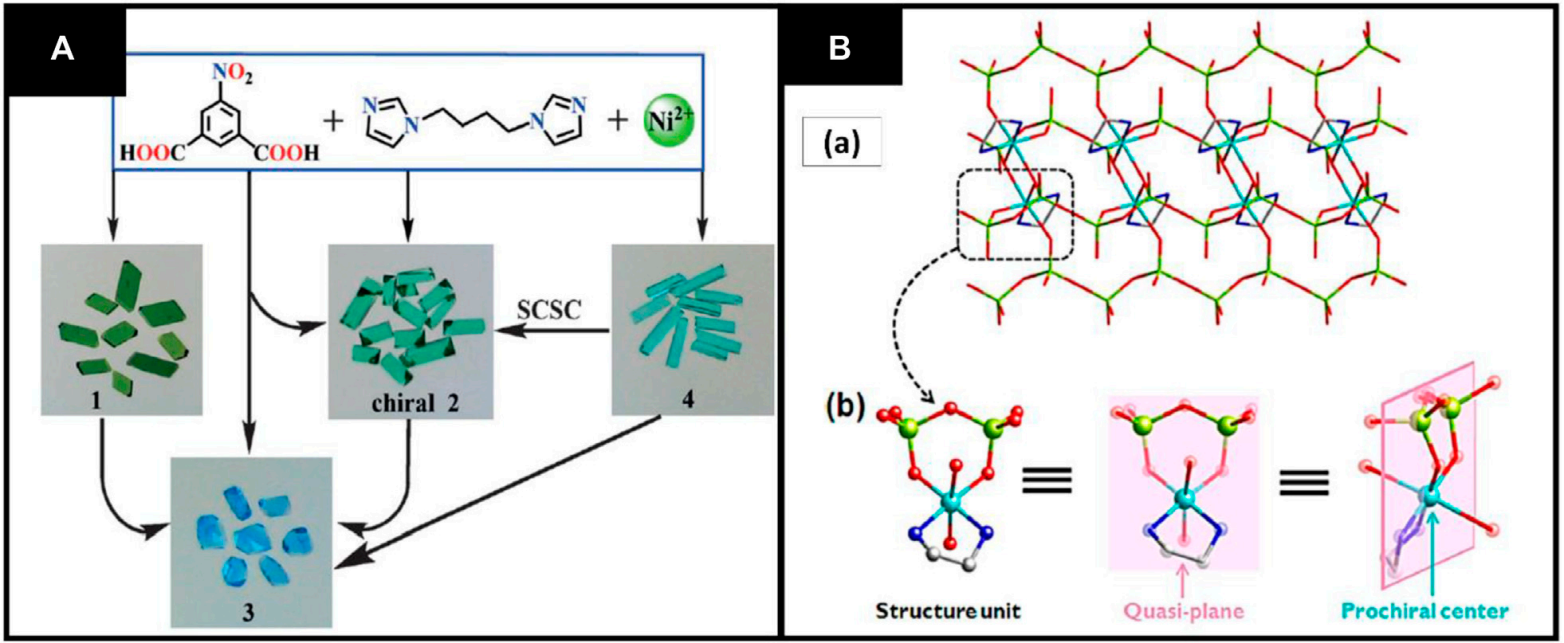
**(A)** Transformation of SCSC 1–4. Reproduced with permission from (Gu et al., 2015) **(B) (A)** Prochiral center and quasi-plane structural unit. Reproduced with permission from (Dhakshinamoorthy et al., 2011b).

Due to many applications of chirality, scientists have been working to convert achiral molecules into chiral ones. In literature, researchers reported many methods for achiral to chiral transformation including use of chiral templates, anionic ions and use of enantiopure ligands (Yi et al., 2012). Recently, Li et al. synthesized achiral to CMOFs by using the solvent-mediated method. In this study, four Ni^+2^ MOFs have been synthesized using 1,4-bis(imidazole-1′-yl)butane (bimb) and 5-nitroisophthalate (NO_2_-ip).These MOFs are [Ni_2_(NO_2_-ip)_2_ (bimb)_1.5_]_n_ (1), [Ni_4_(NO_2_ip)_3_ (bimb)_2_(OH)_2_(H_2_O)]_n_ (CH_3_CH_2_OH)_0.5n_ (2), [Ni(NO_2_ip)(bimb)_1.5_(H_2_O)]_n_ (H_2_O)n. (CH_3_CH_2_OH)_0.5n_ (3), and [Ni(NO_2_-ip)(bimb)(μ-H_2_O)]_n_ (H_2_O)n (4). So, the achiral 4 MOFs transferred to chiral 2 with the help of solvent-mediated method without using any chiral auxiliary, although MOF 3 can be synthesized from 1,2 to 4. The naked eye can see this transformation of CMOFs due to their color change, as shown in Figure 4A (Li et al., 2015).

Before that, Yi and his co-workers synthesized a MOF using achiral to chiral transformation. They used H_6_L as ligand, as building units for construction of new MOFs. The conformational chirality and symmetry breaking in the MOFs were due to this ligand (H_6_L). H_6_L reacts with M^2+^(M = Co., Cd) and results in the formation of two novel porous complexes 1(P-1) and 2 (C2/c). Complexes 1 and 2 react at 100°C with ligand and form two chiral and novel compounds i.e., 3 and 4, respectively (Yi et al., 2012).

#### 2.2.2. Using achiral ligands

Achiral ligands have at least one orientation-reversing isometry and through different engineering approaches, achiral ligands can be used to induce chiral and chiroptical properties (Sato et al., 2020). These ligands have been used in many studies. In 2013, Zhang and his co-workers synthesized 3D chiral MOFs by one-pot solvothermal reaction. In the synthesis imidazole group replaced TPPA, pyridyl group by imidazole group, an asymmetric ligand, MIDPPA, was obtained. Then solvothermal reaction has been carried out using this ligand and Zn(NO_3_)_2_ **·**6H_2_O, 1,2,4-H_3_btc, and an enantiotopic pair 3D CMOF s has been synthesized i.e., 1 L and 1 R, having high e.e. Value. The proportion of 1 L and 1 R is almost 3/1, demonstrating that the solvothermal reaction and asymmetric induced chiral symmetry breaking have occurred. 1 L and 1 R, have ferroelectric, green fluorescent, and second-order NLO properties (Zhang et al., 2017a).

In another study, Dong et al. synthesized CMOFs by prochirality synthetic method using achiral ligands imidazole (Im) and ethane diamine (en) [Cu(en)] [(VO_3_)_2_] (1) was prepared by hydrothermal method using an achiral ligand ethane diamine, which has prochiral center and quasi-helical structure. The other achiral ligand imidazole and compound (1) results in the creation of chiral Copper centers and a CMOFs pair [Cu(en)(Im)_2_] [(VO_3_)_2_] (2a and 2b). This pair of chiral MOFs is one-dimensional and contains helical chains, as shown in Figure 4B (Dong et al., 2014a).

In a nutshell, it should be noted that the chiral inducing agents play a significant role in conversion of non-chiral MOFs to chiral. Variety of these agents are present, including polymers, POMs and ILs, etc. A more striking feature is the size of the CIA, the amount of loading, and its capacity to form interactions (electrostatic, non-covalent, or other), which are the main factors influencing chiral structure.

#### 2.2.3 Asymmetric induction in homo-chiral MOFs

In a study, Wu et al. synthesized two 3D homochiral MOFs by asymmetric induction among two different helices. Compounds 1D (H_3_O)_2_ [Cd_8_ ((R)TMTA)_6_ (bipy)_3_(H_2_O)_4_] and IL (H_3_O)_2_ [Cd_8_ ((S)TMTA)_6_ (bipy)_3_(H_2_O)_4_] were synthesized by solvothermal method using enantiopure ligands (R)-H_3_TMTA and (S)- H_3_TMTA respectively, DMF, Cd(NO_3_)_2_•4H_2_O, triethylenediamine and 4,4′-bipyridine (Wu et al., 2015).

Furthermore, Zhang et al. synthesized CMOFs by using chiral solute and its chirality induction effect. They predicted that using a small amount of alkaloids such as (+)-cinchonine or (−)-cinchonidine can generate a chiral catalytic effect which forms a homochiral metal-organic framework (Me_2_NH_2_)[In(thb)_2_].xDMF (H_2_thb = thiophene-2,5-dicarboxylic acid). This chiral MOF is different because it exhibits permanent microporosity and has a negatively charged framework (Zhang et al., 2008).

## 3 Applications

**FIGURE 5:**
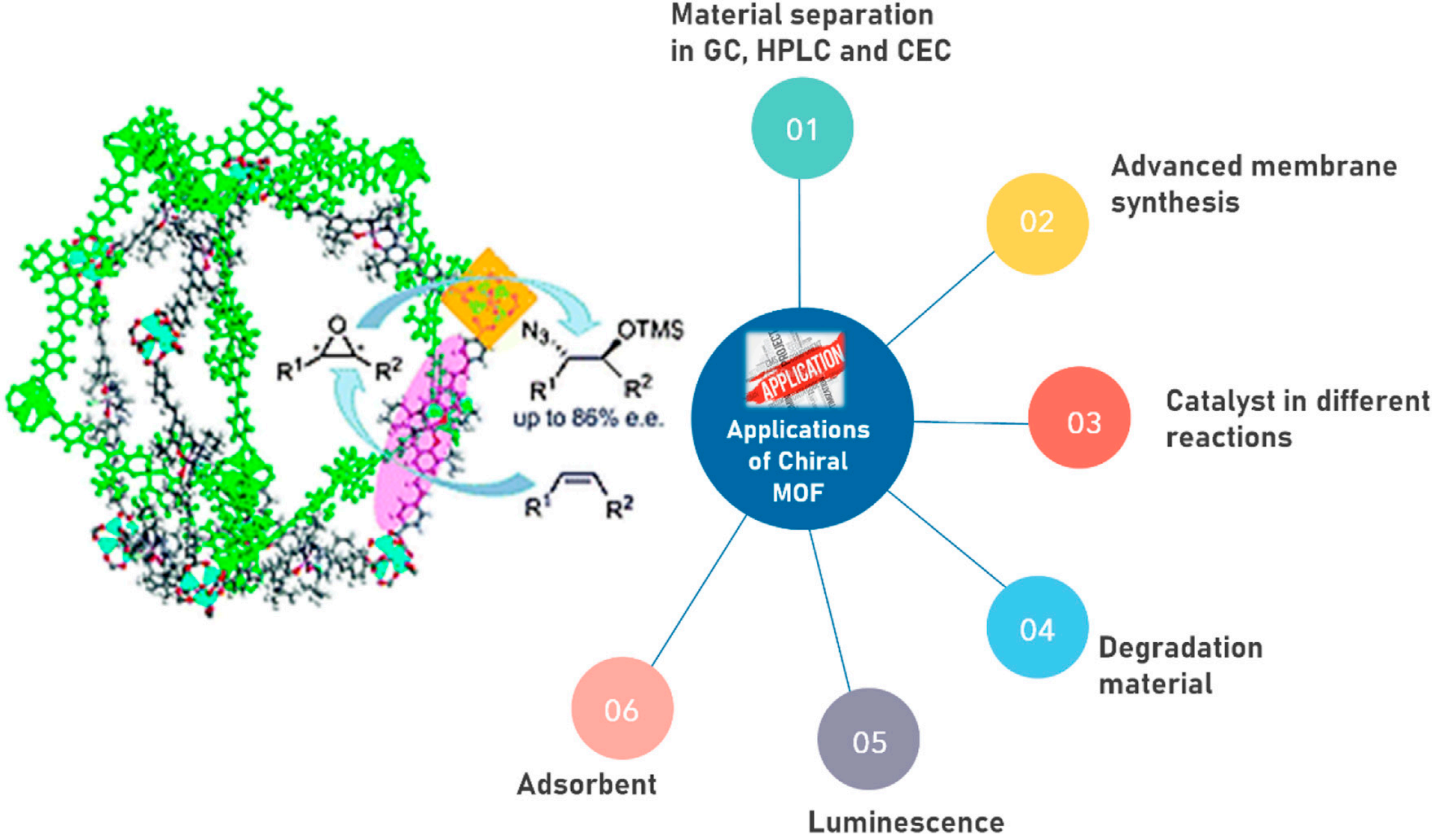
Applications of Chiral MOFs exhibiting multiple advantages of chiral MOFs in many fields of life.

Chirality can be found in nature abundantly and its separation is of great importance as it has applications in the field of molecular biology, pharmacology, drug delivery and chemical compound development. Following the same suit, CMOFs are explored by the scientists and have been prepared to use in multiple fields of life to provide benefits to human due to their excellent properties. A few of such applications are discussed in this paper and shown in Figure 5.

### 3.1 Chiral MOFs as separating material

**FIGURE 6:**
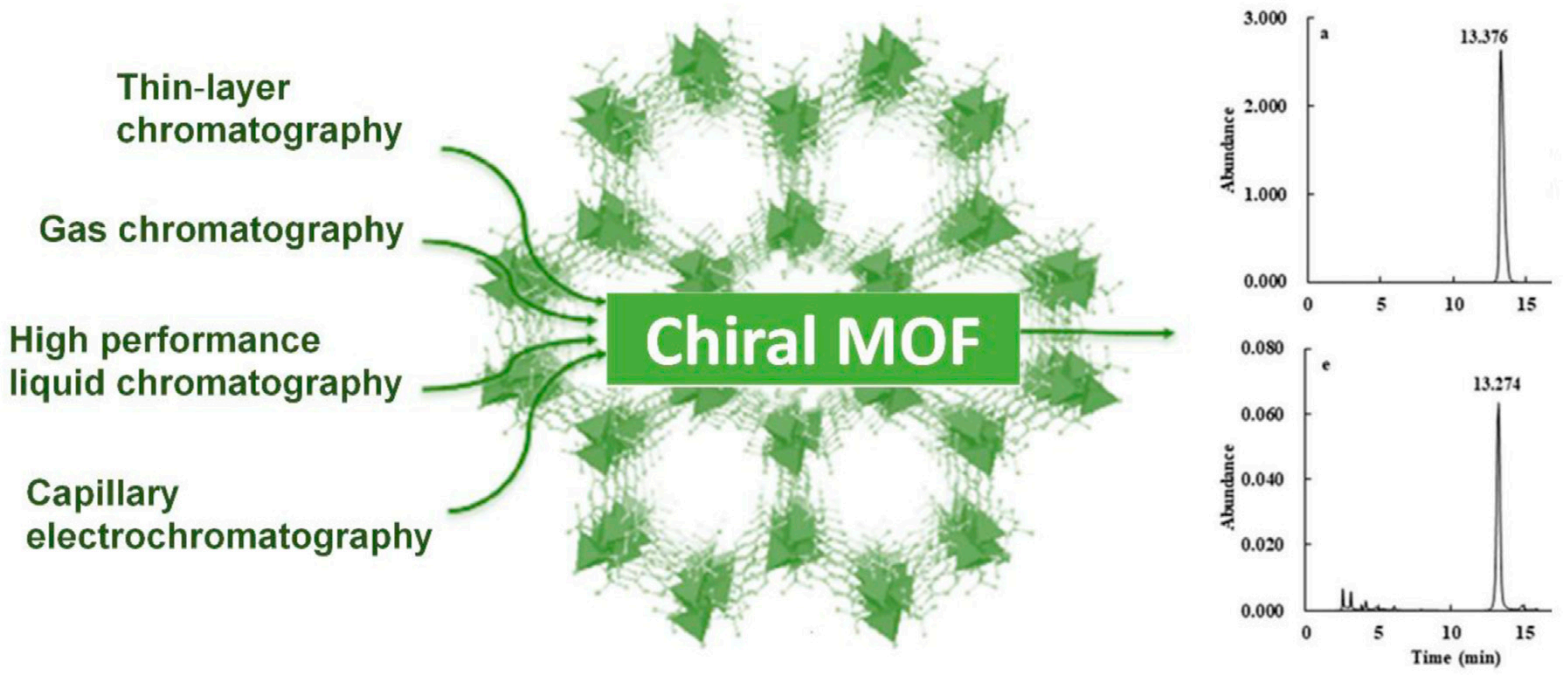
Applications of chiral MOFs in chromatography.

MOFs are built by using metal ions and organic linkers which increases the porosity of the material and amazingly, the level of porosity can be controlled in MOFs which makes them an excellent material for membranes and separation. Functionalized pore surface and larger pore size makes MOFs suitable candidates for separation and storage applications. These tunable properties of MOFs help in chemical separation that was a tiresome task by using the conventional methods used by industries (Karmakar et al., 2017). Schematic diagram of applications of CMOFs in different chromatographic techniques is shown in Figure 6.

Due to the increase in demand for enantioselective separation, variety of CMOFs have been synthesized, which finds applications in HPLC and GC. However, no chiral MOF as stationary phase has been reported for CEC enantioseparation. Cho et al. investigated how post-synthetic modification of MOFs can be done to convert them into optically active materials. They post-synthetically modified, non-chiral coordinatively unsaturated (salen)Zn^II^ compounds with the coordinating enantiopure ligand to produce the enantioselective behavior. So, the complex [bis (pyridine) salen]Zn^II^ was added in presence of Zn^II^(NO_3_)_2_•6H_2_O with biphenyl-4,4′-dicarboxylicacid to create a crystalline framework, which was then developed with ligand, bis [(—)neo-menthol] pyridine-3,5-dicarboxylate.These modified materials had the potential for enantioselective separation and sorption. The preliminary analysis recommends that interacting coordination polymers achieve separation at the surface with the analyte molecules instead of its interior (Cho et al., 2007).

CMOFs have excellent properties like structural uniformity (Goetjen et al., 2020), chemical stability (Li et al., 2017), high surface area (Shen et al., 2020), room for surface modifications (Hou et al., 2018) and large as well as tunable pore size (Gong et al., 2022a). These properties enabled the MOFs to receive attention as a unique media for separation. MOFs are extensively used in HPLC and GC as stationary phase (Yang et al., 2011; Yang and Yan, 2011; Fang et al., 2013). There are multiple benefits of using CMOFs i.e., larger number of elements can be separated (Betzenbichler et al., 2022), the elements with smaller energy differences can be recognized and separated (Betzenbichler et al., 2022), and large flexibility in their designing (Duerinck and Denayer, 2015). The use of CMOFs as a stationary phase is explained in detail for different techniques in following section.

#### 3.1.1 In capillary electrochromatography

**FIGURE 7:**
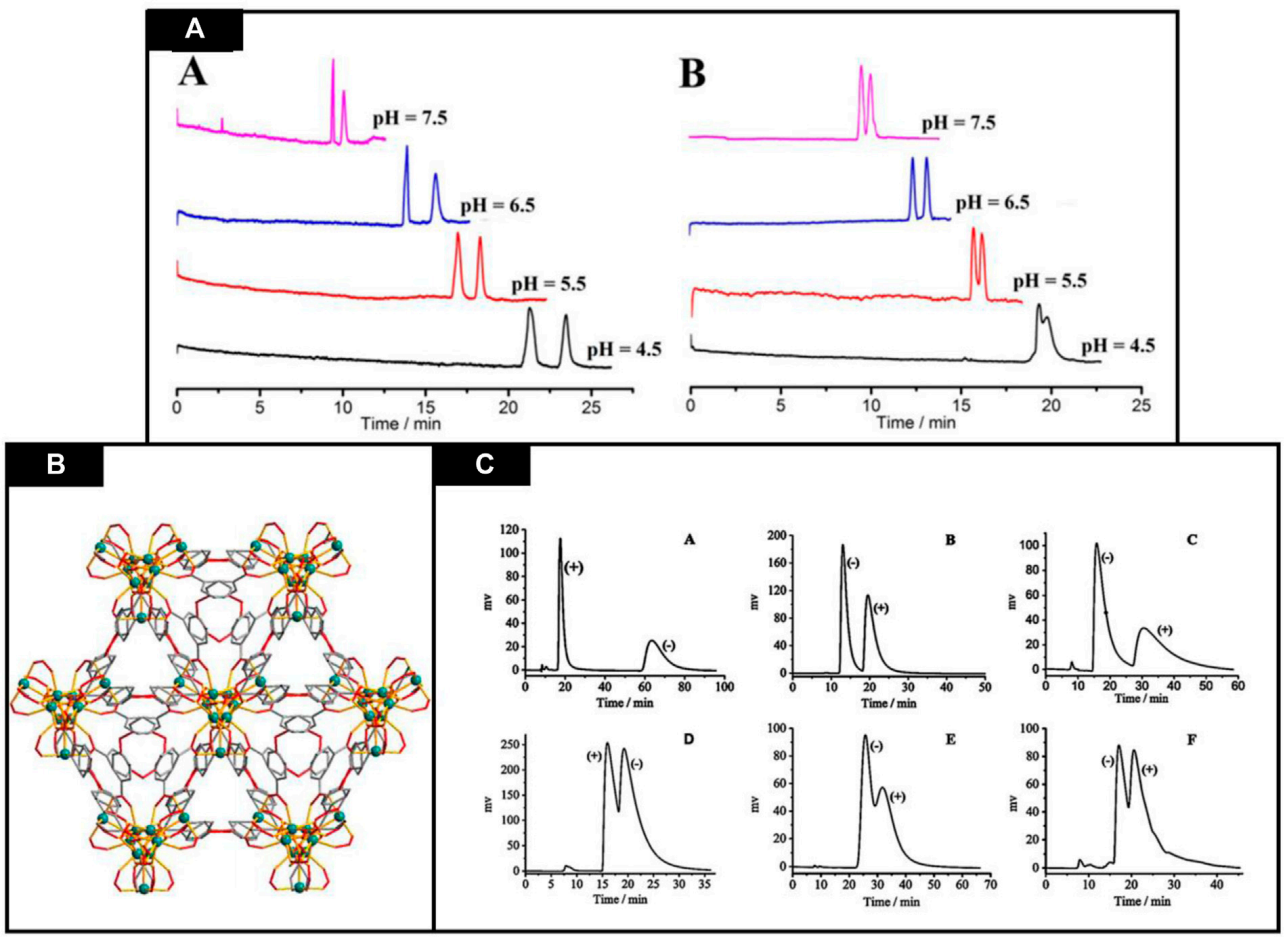
**(A)** Buffer pH effect on flavanone separation **(A)** and praziquantel **(B)**. Reproduced with permission from (Grancha et al., 2017) **(B)** [In_3_O(obb)_3_(HCO_2_)(H_2_O)] solvent 3D structure (O—red In—green,C–gray) Reproduced with permission from (Jeong et al., 2011) **(C)** Racemates separation through Cd-MOF column’ using HPLC. **(A)** 1-(1-naphthyl)ethanol, **(B)** 1,1′-bi-2-naphthol, **(C)** 1-(4-chlorophenyl)ethanol, **(D)** benzoin, **(E)** 1-(9-anthryl)-2,2,2-trifluoroethanol, and **(F)** trans-stilbene oxide. Reproduced with permission from (Zhang et al., 2016a).

CEC is a powerful hybrid analytical technique which utilizes both electrophoresis and chromatography to analyze the molecules under the influence of electroosmotic flow, chromatographic transport mechanisms and bulk movement electrophoretic molecules (Hajba and Guttman, 2021). Currently, many studies have reported the use of chiral MOFs for the sake of chiral separation by electrochromatography. Herein a few studies are explained. Recently, Fei et al. reported a novel, helical and homochiral MOF [Zn_2_ (D-Cam)_2_ (4,4′-bpy)] _n_ (D-Cam = D-(+)-camphoric acid, 4,4′-bpy = 4,4′-bipyridine). This MOF is being applied as a stationary phase in OT-CEC for isomer and chiral composites separation. Using this MOF-coated stationary phase, baseline separation of praziquantel and flavanone was obtained and had a resolution more than 2.10. Effect of change in pH on the separation was also observed and displayed in Figure 7A. With increasing pH from 4.5 to 7.5 migration tendency of both enantiomers increases, and at pH 6.5 greatest resolution has been achieved. So, pH 6.5 was selected for chiral separations. Chromatographs are shown in Figure 5. Besides this, isomers (ionones and nitrophenols) were also separated by using this stationary phase. The standard deviation for retention time, from column-to-column, day-to-day and run-to-run were 3.07%, 2.16%, 1.04% respectively. These results verified that MOFs are potential candidates for the separation of enantiomers in CEC. Hence, by optimization of various parameters i.e. buffer concentration, pH and composition of organic modifier, efficient separation has been achieved. Results indicated that CMOFs are attractive candidates for positional isomers and chiral compounds. These findings paves path for applications of CMOFs in separation (Fei et al., 2014).

In a study, Xie et al. reported the synthesis of a chiral MOF using a solvent with helical structure and countless polytrimer which is presented in Figure 7B. It not only had unique structure but also exhibit excellent thermal, solvent and chemical stability of solvent i.e., [In_3_O (obb)_3_(HCO_2_) (H_2_O)], which makes it a favorable stationary phase for CEC, HPLC and GC. Here, the chiral recognition abilities of this [In_3_O (obb)_3_(HCO_2_) (H_2_O)] solvent has been compared as a stationary phase in GC, CEC and HPLC, respectively (Xie et al., 2014).

#### 3.1.2 In high performance liquid chromatography

The properties like tunable pore size, synthetic conditions and controlling reagents make CMOFs suitable candidate to be used as a stationary phase in HPLC (Liu et al., 2018). There are many studies in literature which indicated the excellent efficiency of the CMOFs in HPLC. Zhang et al., reported a homo-chiral MOF [Cd_2_ (d-cam)_3_].2Hdma·4dma, which can be applied as unique stationary phase for HPLC enantioseparation. Its high surface area, exceptional chemical stability and unique structures make it a promising material for enantioseparation. This MOF also had 6-connected net, and void space in it is much greater than the space in 8-connected BCC net. Due to this structure, sufficient surface area for solute retention had been provided. Chiral compounds separation is a complicated process because of the same chemical and physical properties. In addition, a homochiral MOF having lower column pressure and best selectivity for following racemates enantiomeric separation: ketone, alcohol, aldehyde, base, phenol, a chiral drug, and amide using HPLC was reported. Furthermore, the chiral stationary phase [(CH_3_)_2_NH_2_] [Cd (bpdc)_1.5_] have not been separated from four racemates. Results of this enantiomeric separation indicated that by using this chiral MOF column in HPLC, selective and convenient enantioseparation can be done (Zhang et al., 2014b).

Using this MOF as stationary phase, various racemates were isolated for investigation of chiral recognition ability of the synthesized novel stationary phase. On this Cd-MOF column, following nine racemates were separated: praziquantel, 1-(4-chlorophenyl) ethanol, 1,1′-bi-2-naphthol 1-(9-anthryl)−2,2,2-trifluoroethanol, benzoin, warfarin sodium 1-(1-naphthyl)ethanol hydro benzoin and trans-stilbene oxide. The corresponding retention factors (k_1_′), separation factors (α), mobile phase, and Cd-MOF column resolution for eluted racemates is explained in Table 2 (Zhang et al., 2016b).

**TABLE 2:** Separation of Racemates on Cd-MOF column. Reproduced with permission from (Zhang et al., 2016b).

Sr	Racemates	Mobile phase (v/v)	k_1_′	α	Rs
1	1-(1-naphthyl) ethanol	hexane/DCM (1:1)	1.26	5.7	4.55
2	trans-stilbene oxide	hexane/DCM (1:2)	1.32	1.38	0.59
3	1-(4-chlorophenyl) ethanol	hexane/DCM (1:1)	1.04	2.82	1.26
4	Hydrobenzoin	hexane/DCM (4:1)	1,49	1.28	0.42
5	1-(9-anthryl)-2,2,2- trifluoroethanol	hexane/DCM (4:1)	1.40	1.30	0.52
6	praziquantel	hexane/DCM (2:3)	1.50	1.27	0.46
7	Benzoin	hexane/DCM (1:1)	1.06	1.4	0.62
8	1,1′-bi-2-naphthol	hexane/DCM (2:1)	0.68	2.24	1.76
9	warfarin sodium	hexane/DCM (1:2)	0.31	5.40	0.54

It has been observed that this chiral Cd-MOF column shows baseline separation of 1-(4-chlorophenyl) ethanol and 1,1′-bi-2-naphthol with best peak chromatograms, higher resolution for 1-(1-naphthyl)ethanol (RS = 4.55); meanwhile, 1-(9-Anthryl)-2,2,2-trifluoroethanol, trans-stilbene oxide and benzoin displayed peaks overlap. This behavior has been attributed to tailing, chromatograms enantioseparation which is explained by Figure 7C which indicated that the chiral selector interaction with the help of hydrogen bonding increased peaks tailing because most analytes were chiral and aromatic alcohols.

In another study, [In_3_O(obb)_3_(HCO_2_) (H_2_O)] solvent packed column is applied as stationary phase for separation of several racemates using HPLC. Four types of racemates i.e., omeprazole, furoin, benzamide, 3,5-dinitro-N-(1-phenylethyl) and ibuprofen were eluted using this packed column. Hexane/DCM was used as the mobile phase. Retention factors (k_1_), the efficiency of column (N_1_/m) and mobile phase for the enantiomer which eluted first, resolution (R_s_), and separation factors (α) of the four other eluted racemates are shown in Table 3. Chiral MOF exhibit higher resolution (R_s_ = 1.89) for 3,5-dinitro-N-(1-phenylethyl) benzamide enantioseparation and 4,370 plates m^−1^ column efficiency for furoin, (S)-enantiomer of (Xie et al., 2014).

**TABLE 3:** Different racemates separations on the MOF-packed column. Reproduced with permission from (Xie et al., 2014).

Racemate	Mobile phase	Column efficiency	k_1_’	α	R_s_
Furoin	90:10	4,370	3.25	1.33	1.45
3,5-Dinitro-N-(1-phenylethyl) benzamide	90:10	3,200	0.28	6.08	1.89
Omeprazole	80:10	—	1.91	1.83	0.47
Ibuprofen	90:10	—	1.59	1.92	0.32

A novel homochiral of 3-D MOF (R)-CuMOF-2 was synthesized by Tanaka et al. for the HPLC separation. This MOF had an open cage structure formed by the (R)-3,3′-bis(6-carboxy-2-naphthyl)-2,2′-dihydroxy-1,1′-binaphthyl ligand an initial material. For evaluation of separation behavior of chiral MOF column, twelve racemates were used which included: sulfoxides, 1,3-dioxolan-2-one, sec-alcohols, epoxides, lactone and oxazolidinone. Experimentally it was observed that this homochiral MOF exhibits the best molecular recognition ability so that it can be widely used as stationary phases for enantioseparation (Tanaka et al., 1039).

#### 3.1.3 In gas chromatography

In recent years, the use of MOFs in gas chromatography as a stationary phase had increased by many folds. The 3D framework, high surface area, exceptional thermal stability, homochirality features and absolute helicity makes the MOF column attractive for improved GC separation of the racemates. Many studies have reported an improvement in the separation performance of GC due the effectiveness of MOFs (Firooz and Armstrong, 2022; Zhang et al., 2022). Liu et al., in 2014 synthesized a CMOF to investigate the enantioseparation on CSP in GC. The CMOFs, Co.(d-Cam)_1/2_ (bdc)_1/2_ (tmdpy), was combined with peramylated b-CDs to explore either c-MOF can increase enantioseparations on synthesized stationary phase or not. Incorporating MOF in b-CD column leads to the racemization of GC separation with high column efficiency and enhanced resolution. This research enhanced the chromatographic efficiency of the columns due to better enantioselectivity on GC (Peng et al., 2014).

In another study, [In_3_O(obb)_3_(HCO_2_) (H_2_O)] solvent was used as CSP in GC, and its chiral recognition abilities were investigated by using different racemates. Using MOF-coated capillary column, five racemates were separated, including aspartic acid, limonene, leucine, proline and 1-phenyl-1,2-ethandiol. Retention factors (k_1_), column efficiency (N_1_/m), resolution (R_s_), and separation factors (α) of these five racemates are described in Table 4. Enantioseparation of aspartic acid and 1-phenyl-1,2-ethandiol in GC chromatograms using capillary column coated with MOF had been illustrated in Table 4. For 1-phenyl-1,2-ethanediol high resolution enantioseparation with chromatographic resolution (R_s_ = 1.38), retention time (<1.5 min) and separation factor (α = 1.5) were achieved. Chromatograms observed that the chiral MOF column displayed chiral recognition ability and best selectivity for aspartic acid and 1-phenyl-1,2-ethandiol enantioseparation (Xie et al., 2014).

**TABLE 4:** Different racemates separation on capillary column coated with MOF. Reproduced with permission from (Xie et al., 2014).

Racemate	Temperature (°C)	Column efficiency	k1’	α	Rs
Limonene	125	1,630	1.18	1.35	1.10
1-phenyl-1,2-ethandiol	140	1960	1.17	1.50	1.38
Aspartic acid	120	1870	1.49	1.21	1.12
Proline	100	1,520	1.52	1.30	1.06
Leucine	110	—	2.06	1.05	0.35

### 3.2 Applications of Chiral MOFs in the fabrication of advanced membranes

In recent years, a great progress has been achieved in the field of CMOFs and chiral microporous materials. The membrane technology has revolutionized the fields of chemistry due to a large processing capacity and low-energy consumption and a continuous mode of operation. These membranes have advantages of providing high surface area, varying functionalities, tunable and pore size. Due to multitude of properties, these are widely used in drug delivery systems, chemical sensors, gas storage and catalysis (Lu et al., 2019).

Due to importance of purification and separation of materials in drug development and other applications, engineering CMOFs into membranes is a powerful strategy towards the fabrication of new materials. Many researchers have reported their findings and improved the methods of membrane development which are explained in this review. Kang et al. utilized an *in situ* growth method for the synthesis of homochiral MOF membrane from single nickel source (Kang et al., 2013). Their purpose was to develop a membrane which would be inexpensive, stable at higher temperatures and have simple method of manufacturing. For synthesis, authors used Ni_2_ (L-asp)_2_ (bipy) having Ni (L-asp) layers which was further connected by 4,40 -bipyridine (bipy) linkers for the formation of a pillared structure. Similarly, Li et al. developed [Ni_2_(mal)_2_ (bpy)].2H_2_O (Ni-MB) chiral MOF membrane which was synthesized using secondary growth and high ball milling and found to be suitable for the separation of chiral molecules (Li et al., 2016).

In another study, Liu et al. fabricated a nanosheet of exfoliating layered CMOFs. In this study, Ln-MOFs were used for the synthesis which were based on a 1,1′-biphenyl skeleton. These skeletons were having pendant mesityl groups at the 3,3′-position. These membranes were studied to detect the terpenes and terpenoids and results indicated their excellent selectivity and sensitivity (Liu et al., 2021). Peng et al. developed CMOF based membranes which had chiral -methoxy auxiliaries or dihydroxy auxiliaries that were obtained from manganese carboxylate chain and enantiopure tetracarboxylate-bridging ligands of 1,1′-biphenol (Peng et al., 2014). These membranes had high enantioselectivity which helped in material absorption and separation.

The application of CMOFs in material separation is significantly important and improved the processing capabilities of systems due to the fact that these can be tailored and tune according to the required chirality and porosity (Xue et al., 2016). Although, there are numerous studies which have proven their efficiency and suitability, the combination of porosity with chirality is a difficult milestone to achieve.

### 3.3 Chiral MOFs as catalysts

In the past decade, CMOFs have been seen as a heterogenous catalyst in various applications. It possesses a wide range of properties which makes it suitable to be used in multiple types of reactions including photocatalysis based reactions. The details of use of CMOFs in different types of reactions are discussed here.

#### 3.3.1 Asymmetric catalysis

**FIGURE 8:**
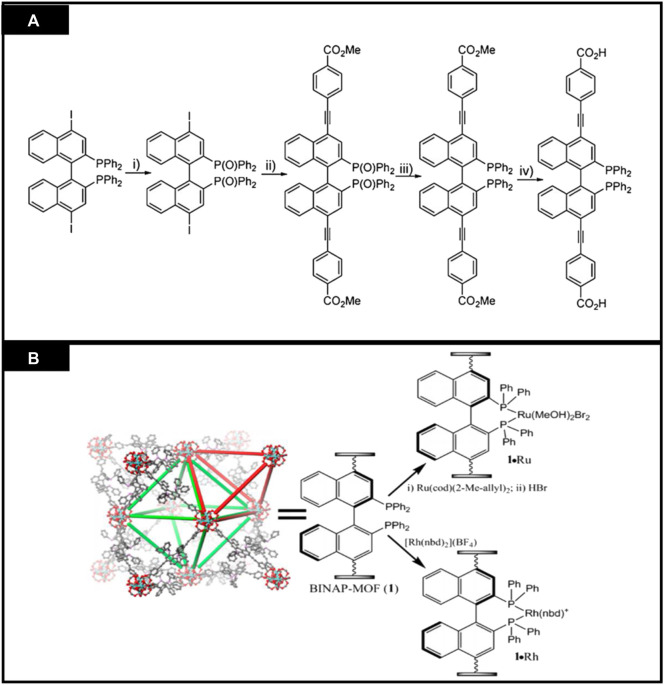
**(A)** Synthesis of a 2,2′-bis(diphenyl phosphino)-1,1′-binaphthyl(BINAP)derived dicarboxylic acid ligand **(B)** Post modification of BINAP-MOF with metals. [Reproduced with permission from Falkowski et al. (2014)].

CMOFs act as heterogeneous catalysts for several organic reactions as solid catalysts. In the enantioselective reactions, these reactions cannot be completed by using traditional heterogeneous catalysts. Their reusability, stability and porosity make them attractive chiral catalysts for organic transformations. CMOFs are used as catalysts in some novel enantioselective reactions and many studies have reported their successful synthesis and application. For example, Lin et al. studied the catalytic activities of homochiral metal-based MOFs for a variety of asymmetric organic transformations (Falkowski et al., 2014). Ruthenium and Rhodium-complex-based on BINAP-derived CMOFs were highly enantioselective for asymmetric reactions. BINAP-derived from dicarboxylic acid, H_2_L^12^, was synthesized by multiple-step reaction which starts from 4,4′-I_2_-BINAP (Figure 8). Chiral Zirconium MOF was synthesized from H_2_L^12^ and [Zr_6_O_4_(OH)_4_ (L^12^)_6_] was used for its post-synthetic metalation, then treated with [Rh (nbd)_2_(BF_4_)] to form [Zr_6_O_4_(OH)_4_ (L^12^)_6_].Rh and also with Ru (cod)(2-Me-allyl)_2_ and HBr to form [Zr_6_O_4_(OH)_4_ (L^12^)_6_]·Ru. Synthesis is shown in Figures 8A,B. For 1,4-addition of aryl boronic acid to 2-cyclohexanone, 1 mol% catalyst was used, showing up to 99% enantioselectivity. Similarly, the same catalyst showed excellent activity for 1,2-addition of AlMe_3_ to α, β-unsaturated ketone to form chiral allylic alcohols. MOF functionalized with Ru exhibit outstanding activity in substituted alkenes and *β*-keto ester hydrogenation, having ee up to 91% and 97% respectively.

**FIGURE 9:**
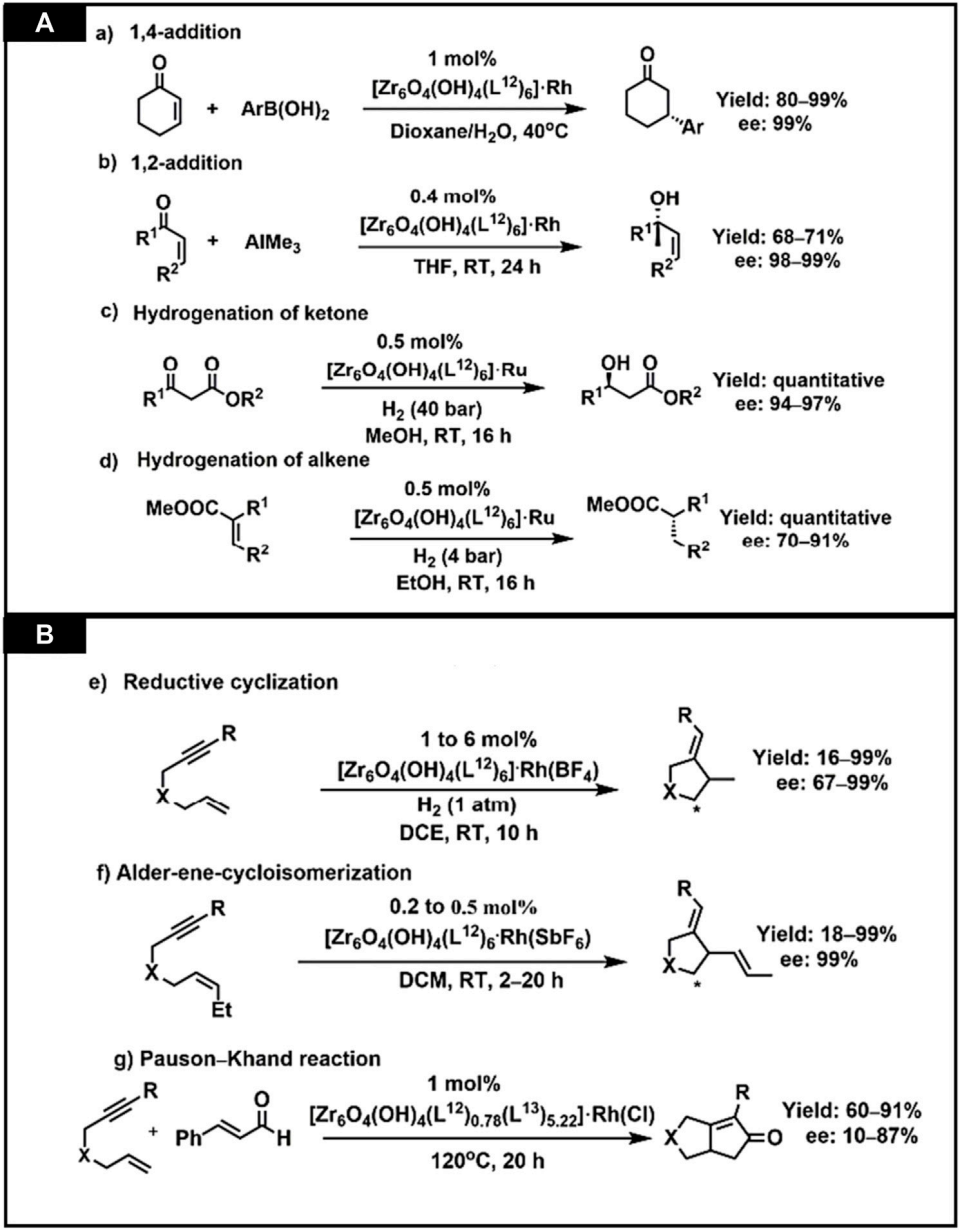
**(A)** BINAP-MOFs catalyzing asymmetric organic transformations **(A)** 1,4-additionreaction by MOF(25a), (**(B)** 1,2-addition reaction by MOF (25a), **(C)** Hydrogenation of ketone byMOF (25b), (**(D)** Hydrogenation of alkene by MOF (25b) **(B) (E)** Reductive cyclization of 1,6-enynes,**(F)** Alder-ene-cycloisomerization of 1,6-enynes, **(G)** Pauson-Khand reaction catalyzed by MOF (26a). Reproduced with permission from (Bhattacharjee et al., 2018).

Later, the scope of the BINAP-MOF was extended by the same group (Sawano et al., 2015). Rh catalyst exhibits good yield and enantioselectivity up to 99% for Alder-ene cyclo isomerization of 1,6-enynes to construct cyclic products and asymmetric reductive cyclization. These catalysts show 4 to 7 times higher enantioselectivity and catalytic activity than conventional catalysts. Though, no catalytic recyclability was observed from the recovered BINAP-MOF Rh catalyst. One of the drawbacks of this study was that this catalyst did not show any results for asymmetric Pauson–Khand-type reactions which were sterically hindered. To solve this issue, Lin and his co-workers presented a concept known as mixed ligand concept. According to this concept the area close to the catalytic sites can be expanded and intermediates of reaction can be accommodated, and they successfully explained Pauson–Khand-type reactions having ee of 87%. The chiral MOF [Zr_6_(OH)_4_O_4_ (L^12^)_0.78_ (L^13^)_5.22_](**26**)(H_2_L^13^ = 4,4′-[(2-nitro-(1,1′-binaphthalene)-4,4′-diyl]bis (ethyne-2,1-diyl)dibenzoic acid with mixed linkers was reacted with [RhCl(nbd)]_2_ to form [Zr_6_(OH)_4_O_4_ (L^12^)_0.78_ (L^13^)_5.22_].RhCl (26a). The catalytic activity of this MOF (26a) was 10 times more than the control catalyst, which is also homogeneous catalyst. Figures 9A,B elaborates the catalytic activities of synthesized MOF for organic transformation reaction (Bhattacharjee et al., 2018).

#### 3.3.2 Asymmetric epoxidation reaction

Asymmetric Epoxidation Reactions converts the allylic alcohol into an epoxy alcohol. CMOFs has been extensively used in these reactions to synthesize membranes. In this regard, Song et al., reported two novel CMOFs (1 and 2) which were synthesized by using the Mn-salen-derived dicarboxylate-bridging ligands (L4-H2 and L3-H2). Moreover, the secondary building units were [Zn_4_ (m_4_-O)(O_2_CR)_6_] and [Zn_5_(H_2_O)_2_ (m_3_-OH)_2_(O_2_CR)_8_] respectively. These MOFs has an application in sequential catalysis of regioselective epoxide ring-opening reactions and stereoselective.

These MOF-catalyzed reactions gave moderate ee and excellent yields as compared to homogeneous controls, but selectivity and activity remain the same. Alkene epoxidation.2-(t-butylsulphonyl)iodosyl benzene were used as oxidant and the aromatic substituted olefins were taken as substrates to check the catalytic activities of both MOFs i.e., CMOF-1 and CMOF-6, for asymmetric epoxidation reaction. Based on proposed structures of polymorphs and TGA solvent weight loss, CMOF-**1** has an open channel of smaller sizes and is less porous than CMOF-**6,** but CMOF-**1** catalytic activity is high for asymmetric epoxidation. The [Zn_4_ (m_4_-O)(O_2_CR)_6_] nodes present close to the other network make catalytic active Mn-salen sites of CMOF-**6** less available to substrates. This hindrance consequently lowers catalytic activities of Mn-salen sites in CMOF-**6**. It is proposed that [Zn_4_ (m_4_-O)(O_2_CR)_6_] nodes catalyzed the ring-opening reaction of epoxide without interfering Mn-salen centers. Therefore, the CMOF series act as catalysts for epoxide ring-opening and sequential reactions of alkene epoxidation. Both CMOFs exhibited catalytic activities and give the product of ring-opening having significant ee values and yields. Out of all possible four pairs only one pair of enantiomers was detected, and ees value of the products of ring-opening reactions were the same as corresponding epoxides, which shows that this reaction exhibit f high stereo- and regio-selectivity (Song et al., 2012).

#### 3.3.3 Asymmetric transfer hydrogenation of amines

CMOFs have been considered a fascinating material because they act as heterogeneous catalysts for asymmetric transformation reactions. Li et al., computationally designed two Zr-based CMOFs (-bct and-fcu) by decorating zirconium clusters with organic linkers derived from phosphoric acid. DFT method was used to check the ATH of N, 1-diphenylethan-1-imines calculated the catalytic activity of these two MOFs. Transition states of both MOFs (E)-syn-TS-S and (Z)-syn-TS-R makes hydrogen bonds with chiral active sites. Gibbs energy of (E)-syn-TS-S is higher than (Z)-syn-TS-R, but the mechanism *via* (Z)-syn-TS-R is thermodynamically and kinetically suitable. The ee values to form (S)-/(R)-amines were proposed to be 96.6% on MOF-bct and 99.9% on MOF-fcu, respectively. Although in a kinetically favored pathway, ee value is zero for ATH, which is catalyzed using chiral phosphoric acid (L_1_A_2_). The extraordinarily increased enantio-selectivity on MOFs is endorsed to confinement effect and steric hindrance of framework cavity. Ee value of MOF-fcu is higher than MOF-bct because MOF-fcu has a 12-connected octahedral cavity that shows improved confinement compared to a 10-connected octahedral cavity in MOF-bct.

Additionally, some solvents (acetonitrile, toluene and dichloromethane) are studied for ATH. By lowering solvent polarity, enantioselectivity remains almost unchanged, but the activation barrier drops. Toluene seems to be the best among these solvents. The computational study gives microscopic insight into the imine transformation mechanism on the CMOFs and explains the role of shape and cavity size in transformation. These two CMOFs exhibit higher enantioselective performance to be used for ATH of imines. This study unveiled that cavity and framework topology play an important role in enantioselectivity. Hence, enantioselectivity can easily be tuned by tailoring cavity and topology which cannot be gained on homogeneous catalysts. The bottom-up insight and synthesis strategy of this work open up pathway for development of novel CMOFs for efficient asymmetric transformations. (Li and Jiang, 2019).

Chen and his coworkers rationally designed CMOFs by sterically demanding groups as ligands and higher electronegative atoms as metal ions. Strontium, zinc and calcium based CMOFs were synthesized by using 1.1′-biphenol enantiopure phosphono-carboxylate ligands which are later functionalized at 3,3′ position. The metal phosphonates are uniformly distributed along channel of CMOFs, which act as Lewis acids and help to catalyze ATH of heteroaromatic imines (quinolines and benzoxazines). Among all the catalysts, calcium based CMOFs substituted with trimethyl exhibited highest enantioselectivity and catalytic activity. This is first attempt to use non-noble metal phosphonate active sites for ATH reactions by using Hantzsch ester as source of hydrogen. This stud y opened pathway for synthesis of variety of CMOFs for asymmetric application (Chen et al., 2021).

#### 3.3.4 Grignard type reactions

**FIGURE 10:**
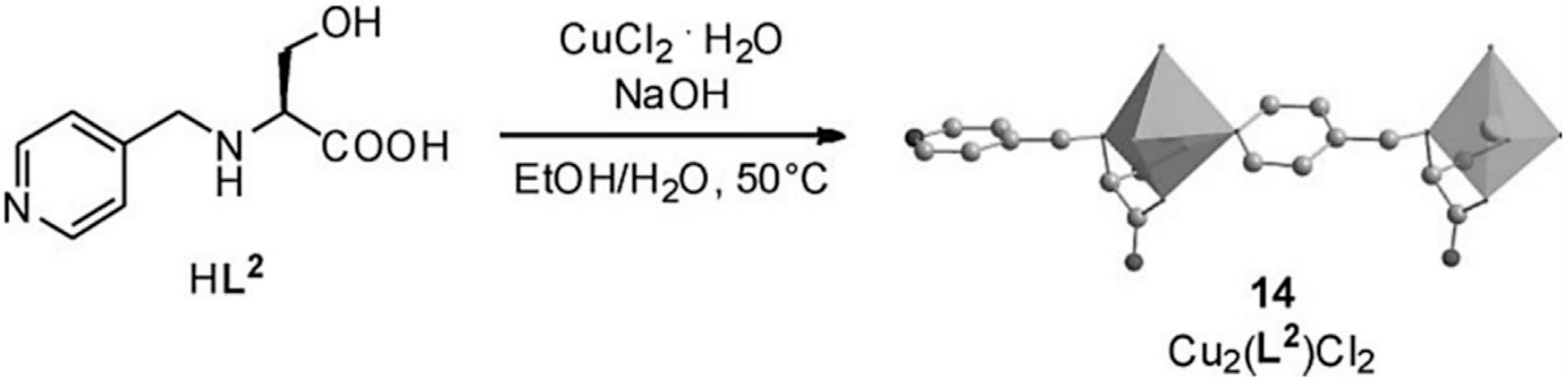
**(A)** The reaction and crystal structure of MOF 14. Reproduced with permission from (Sato et al., 2020).

CMOFs can be used for asymmetric catalysis of Grignard reactions. Homochiral solid Cu_2_(L_2_)_2_Cl_2_ is synthesized by reaction of HL_2_ with CuCl_2_ in ethanol/water solution as shown in Figure 10A. In 2009, L-serine, a chiral amino acid, gave a linker that is also chiral i.e., HL_2_, by functionalization with pyridine substituent.

In MOF 14, two crystallographically different Cu atoms are present (14). Each Cu atom in the framework was linked to an amino group, a hydroxy group, and a carboxylate group from one L_2_ and second L_2_ by pyridyl group. This coordination geometry has an important feature: the amino group’s N atom has some information about chirality. This chirality was introduced by chiral carbon of neighboring atoms and stabilized by Cu interaction. Moreover, one Cu atom in the framework is also coordinated by two Cl atoms and the second atom of Cu was coordinated with one Cl atom, which results in octahedral and square pyramidal geometry, respectively. The coordination results in the formation of a framework having 1-dimensional chains. One of the Cl atoms helped in interconnecting these chains, which forms bilayers and runs along the different orientations. A framework containing one-dimensional channels with a size of 5.1 Å × 2.9 Å is formed by supramolecular interactions of bilayers. However, for the substrate of test reaction, these channels are too small i.e., cyclohexlmagnesium chloride addition to different *a*,*b*-unsaturated aldehydes. Hence, this reaction takes place on the outer layer of the framework, than inner channels. Although, good to excellent ee values i.e., 99% and high conversions of 93%, were achieved for a reaction between cyclohexylmagnesium chloride and 4-(4-methylphenyl) but-3-en-2-one in solvent THF. Additionally, framework (14) also showed more enantioselectivity at a lower rate of conversion than its analogous homogenous catalyst HL_2_ in the reaction between 4-phenylbut-3-en-2-one and cyclohexylmagnesium. Similarly, (14) shows that the chiral alcohol had 88% ee and 48% conversion, although HL_2_ exhibits a lower ee value of 51% and higher conversion (Table 5). According to C.D. Wu et al., this behavior was attributed supplementary chiral information present on the amino group of MOF (Wang et al., 2009). The heterogeneous character of catalyst was proved by filtration (Nickerl et al., 2011).

**TABLE 5:** Grignard reactions catalyzed by MOF 14. Reproduced with permission from (Nickerl et al., 2011).

R1	R2	Catalyst	Conversion (%)	ee (%)
Me	Me	14	93	>99
Cl	Me	14	94	97
H	H	14	88	55
H	Me	14	97	65
H	Me	HL^2^	84	51

So, CMOFs are potential candidates for asymmetric heterogeneous catalysis. Besides high yields, ee values of CMOFs surpass homogeneous counterparts. Apart from this MOFs exhibit feature to form size selective effect on the substrates which are converted, it cannot be done by homogeneous catalysts. Till now, the catalytic activity of CMOFs catalysts is attributed to functional groups present at linker moieties or molecules introduces post-synthetically.

### 3.4 Chiral MOFs as degradation material

Organic azo pollutant degradation by the Fenton oxidation process is a well-known process, but the problem is that the reaction rate is slow at an ambient temperature. It is mentioned in the literature that a mixture of Congo red and H_2_O_2_, a well-known water pollutant, has low degradation efficiency i.e., 13% and is stable up to 130 min. The degradation mechanism illustrates that metals that have variable oxidation are efficient catalysts for degradation.

Bhattacharya and his co-workers used coordination polymers to enhance the degradation rate. They synthesized a ligand (TPMP) by reacting 3-amino-5-methyl-1H pyrazole and terephthloyl chloride, in trimethylamine presence. The reaction was performed under an inert atmosphere, and acetonitrile was used as a solvent.

They synthesized four different MOFs of Co., (II) using this ligand and these MOFs were used for degradation. In this process, hydroxyl radicals were generated under UV light in Co., (II) MOF presence. The chiral MOF acts as an oxidant, and it readily decomposes organic pollutants. Following is the mechanism for an azo dye degradation.

$$Co\left(II\right)\;MOF+H_{2}O_{2}\;\Rightarrow Co\left(III\right)\;MOF+OH+OH^{-}$$

$$Co\left(III\right)\;MOF+H_{2}O_{2}\;\Rightarrow HO_{2}+Co\left(II\right)\;MOF+H^{+}$$

Azo  dye+OH ⇒ Oxidation  Products

These compounds act as heterogeneous catalysts because they are water insoluble. The photocatalytic activity was challenging to perform with MOF-1 because it loses its crystallinity at room temperature. The other three MOFs were used in the presence of H_2_O_2,_ and the experiment was performed with UV-Vis. It was observed that CR solution absorption decreases with consistent intervals. Bleaching of red color dye also reveals a degradation process. No decrease in absorbance was observed in the control experiment, which shows that reactions do not proceed without catalyst. The degradation efficiencies are 83.19% for MOF-2, 51.66% for MOF-3, and 85.62% for MOF-4, and for a control experiment, it was just 16.03%. Kinetics studies showed that pseudo-first-order was followed by the reaction (Bhattacharya et al., 2016).

### 3.5 Chiral MOFs as catalysts

**FIGURE 11:**
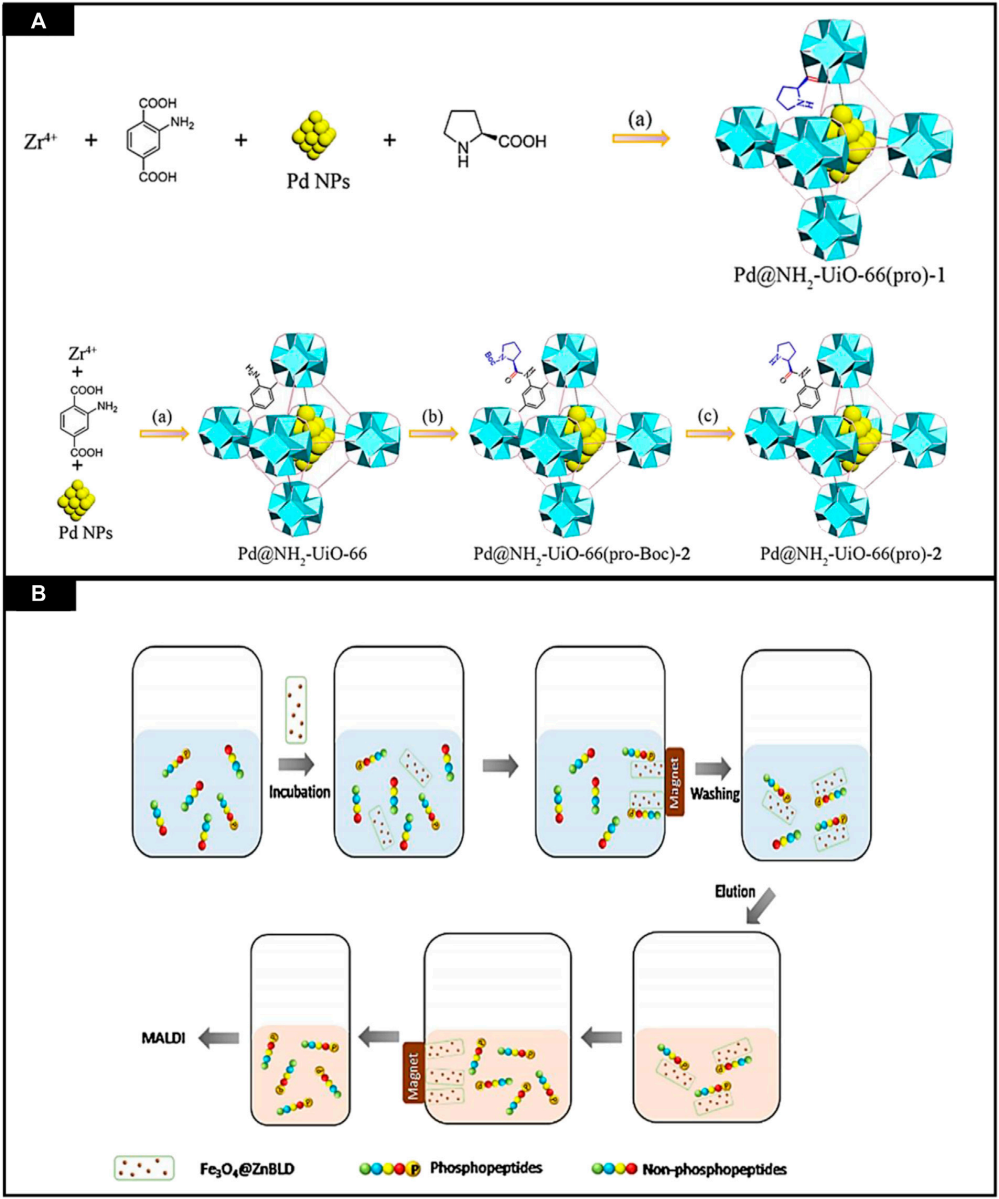
**(A)** Synthesis of Pd@NH_2_-UiO-66 (pro)-1 and Pd@NH_2_-UiO-66 (pro)-2 catalyst. Reproduced with permission from (Wu et al., 2015) **(B)** Phosphopeptides selective capturing by using Fe_3_O_4_@ZnBLD composites. Reproduced with permission from (Gu et al., 2016).

The development and design of multifunctional catalysts can be used for sequential reactions which is a significant challenge. Cheng et al. reported the bifunctional metal NPs@CMOFs as catalysts for the asymmetric sequential reactions. For the construction of two chiral and bifunctional catalysts, chiral proline and palladium nanoparticles were added to NH_2_-UiO-66. In it “bottle-around-ship” method was used for encapsulation of active palladium nanoparticles in frameworks. By post-synthetic modification of organic linkers and coordination to zirconium nodes, chiral proline was added in NH_2_-UiO-66, as shown in Figure 11A. This chiral proline-with bifunctional Pd@NH_2_-UiO-66 catalysts was used for asymmetric aldol reactions and sequential Suzuki coupling, having good enantioselectivities, i.e., ee anti values up to 97%, exceptional performance in coupling and had 99.9% yield. This heterogeneous catalyst can be reused, and the important thing is that the reaction activity was not lessened even after four cycles (Cheng et al., 2020).

### 3.6 Chiral MOFs as capturing material

MOFs are considered porous materials, having tunable structures and high surface area. Moreover, their surface may be modified, and functionalization can also be performed. In 2013 MOFs were used for selective enrichment of phosphorylated peptides (Messner et al., 2013). Zhao et al. designed a MOF for research on phosphoproteome (Zhao et al., 2014). Although, these magnetic MOFs were not very specific in their activities. Herein, 3D homochiral MOFs were synthesized and then decorated with magnetic nanoparticles by facile reaction and used to capture phosphopeptides in intricate biological samples with high selectivity and efficiency. Fe_3_O_4_@ZnBLD was synthesized and then utilized for the enrichment of phosphopeptides. The enrichment process employed is shown in Figure 11B.

To evaluate the performance of MOFs, standard phosphoprotein, and β-casein digest was selected as standard. This phosphoprotein was incubated with Fe_3_O_4_@ZnBLD in loading the buffer. After this, MOF was segregated from the mixture using a magnet and lavation was done thrice using the same buffer. NH_3_·H_2_O (10%) was used for elution of captured phosphopeptides and then analyzed by using MALDI-ToF MS. By using this buffer, several non-identified peaks appeared in mass spectra so, the composition of loading buffer was changed, and 500 μg of MOF composite was considered an optimum amount to obtain an average signal intensity of the detected phosphopeptides.

**FIGURE 12:**
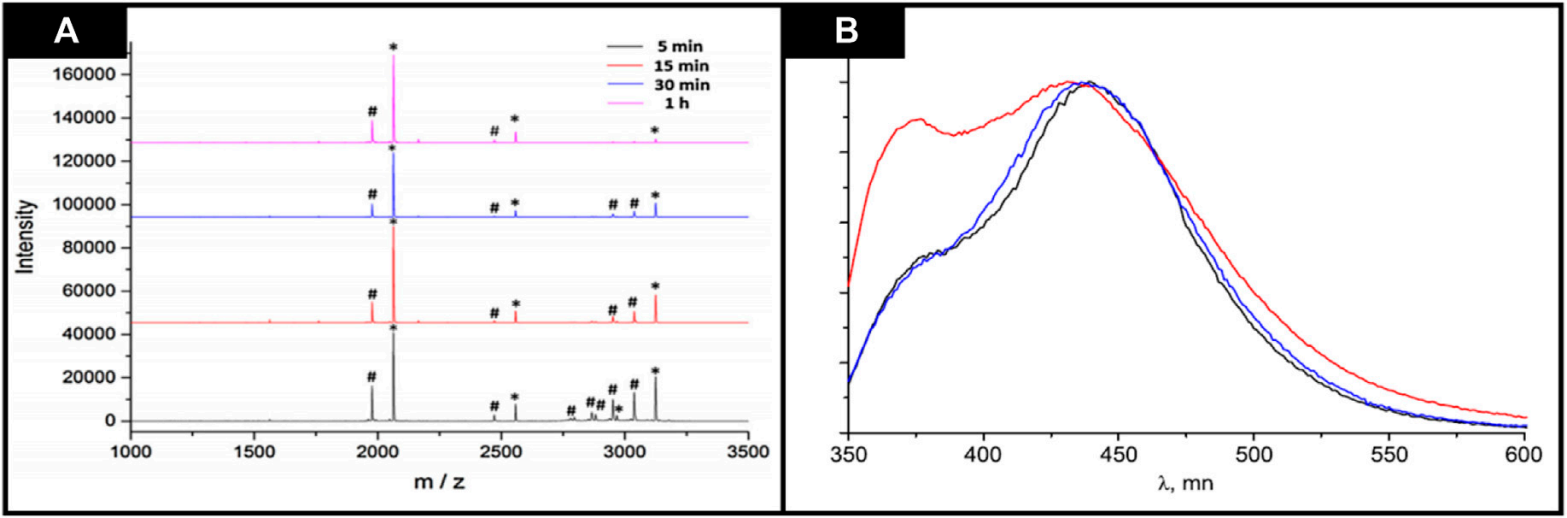
**(A)** Incubation time optimization for phosphopeptides enrichment in2 × 10–^7^ M–casein by using Fe_3_O_4_@ZnBLD composites (“*” shows phosphopeptides and “#” shows dephosphorylated peptides after HPO_3_ loss). Reproduced with permission from (Gu et al., 2016) **(B)** Emission spectra (330 nm) of [Zn_2_ (dmf)(bdc)(S-lac)] DMF Reproduced with permission from (Zhang et al., 2017b).

The phosphopeptides were captured because they can bind with phosphate moieties and ions of metals by coordination bonding and electrostatic interactions. Moreover, some theoretical calculations also showed that the MOFs possessed affinity properties for phosphopeptides. Performance of Fe_3_O_4_@ZnBLD composites was evaluated by increasing incubation time from 5 to 60 min. Figure 12A showed the results that illustrate that the intensity of mass spectra signals increased when the incubation time was increased, but the intensity was declined after 1 h. This behavior was attributed to competitive adsorption of the non-phosphorylated peptides. So, the enrichment process took 10 min which was followed by MALDI-ToF analysis. This work has opened the pathway for synthesizing new chiral materials and using them as affinity hook for capturing peptides in the research (Qi et al., 2016).

### 3.7 Chiral MOFs as luminescent material

A porous homochiral MOF consists of two organic ligands, one structurally rigid and another one chiral and metal cations. The prototype of family is [Zn_2_ (dmf)(bdc)(S-lac)] zinc (II) lactate terephthalate, and it bears chiral S-lactate as its centers, which are decorated by networks of ca (5 Å), made by the terephthalate spacers, which upon photoexcitation emits luminescence of violet-blue color. [Zn_2_ (dmf)(bdc) (S-lac)] separated chiral alcohols and sulfoxides by intermolecular interactions, which are stereospecific, between framework atoms and chiral isomer. So, all chiral isomers should show a distinctive influence on the electronic structure and geometry of the host, it certainly affects the host’s luminescence properties. Keeping this point in mind, Zavakhina et al., in 2019, investigated homochiral and porous framework [Zn_2_ (dmf)(bdc)(S-lac)] and its host–guest chemistry of with S-or R-1,2-propanediol by using single-crystal XRD. These two novel inclusion compounds were obtained by dipping [Zn_2_ (dmf)(bdc)(S-lac)]. DMF in neat S-1,2-propanediol (S-pd) or R-1,2-propanediol (R-pd). Single crystal XRD analysis of [Zn_2_ (dmf)(bdc) (S-lac)] _S-pd, and [Zn_2_(S-pd)_2_ (bdc) (S-lac)] _R-pd indicated that these two enantiomers are of similar alcohol, but their reaction with the chiral and porous framework was different, because of different interactions with the host and different positions. S-pd enantiomer linked to Zn cations of porous framework and replaced both coordinated DMF and guest and of real MOF, but the R-pd only behaves as guest molecule inside channels. Host [Zn_2_ (dmf) (bdc) (S-lac)] luminescent properties were affected by guest molecules (1,2-propanediol) chirality. In [Zn_2_ (dmf) (bdc) (S-lac)].R-pd luminescence spectra a new peak was observed in comparison with original host while the [Zn_2_ (S-pd)_2_ (bdc) (S-lac)].S-pd luminescence spectra was identical to original spectra of [Zn_2_ (dmf) (bdc) (S-lac)].DMF. So, the chiral guests incorporation enhanced the luminescence quantum yields of R-pd (u = 66%) and S-pd (u = 34%) compared to [Zn_2_ (dmf)(bdc)(S-lac)]. The emission spectrum is shown in Figure 12B. DMF (u = 21%). This behavior showed stereoselective sensing by the porous and homochiral coordination polymer, a highly desired but sporadic phenomenon for the development of chiral sensors (Zavakhina et al., 2019).

Gao et al., in 2022 used the reticular chemistry to construct CPL materials. Construction of CPL materials with high rigidity and porosity is very challenging but homochiral porous MOFs solved this challenge. Enantiomeric pair of MOFs has been synthesized by employing achiral luminescent ligand TPB and D/L-cam. Hierarchical chirality leads to higher CPL activity, two homochiral MOFs Zn-CMOF-L and Zn-CMOF-D exhibit same space groups and their CPL activity do not follow total structure chirality. Moreover, single phase white emission was achieved by encapsulation engineering of MOF⊃dye host–guest. In this work An-CMOF-D⊃AO act as phosphors and produces single phase white light emission. This work paves path and encourages chemists to exploit field of chiral luminophores (Gao et al., 2022).

### 3.8 Chiral MOFs as adsorbents

In the field of chemistry, chiral molecules enantiomer separation is very significant, and has several applications in the field of agriculture, pharmaceutics and chemical engineering. MOFs are favorable materials for the efficient separation of enantiomers due to their crystalline structure and large surface area. In 2015 Gu et al. investigated the pore size effect of isoreticular CMOFs on the property known as enantioselectivity. So, the enantioselectivity was studied during chiral molecules adsorption, (S)- or (R)-limonene, in homochiral and pillared-layer MOFs of Cu_2_(Dcam)_2_(L)^28^ type, which had a different type of pillar linkers L but the identical type of chiral linker (1R, 3S)-(+)-camphoric acid (Dcam) layer. The pillar linkers N are L-donor ligands of type 1,4-bis(4-pyridyl)benzene (BiPyB) and diazabicyclo [2.2.2]octane (dabco),4,40-bipyridyl (BiPy). Copper complexes coordinated these linkers at an axial position, perpendicular to chiral Cu_2_(Dcam)_2_ layers, forming pillars of different lengths. They had a pore size of 0.4, 0.8, and 1.2 nm respectively, in [001] direction and 0.7 nm in [100] and [010] direction. For performing adsorption experiments effectively, thin films of MOFs were being used. These thin films are named as SURMOFs. It was observed that by increasing pore size, adsorption capacity also increases and for enantiomer selectivity, SURMOFs having medium pore size showed the highest enantiomeric excess, although SURMOFs with large and small pores showed small enantiomeric excess. This study revealed that pore size should also be adjusted (Gu et al., 2015).

### 3.9 Chiral MOFs as sensors

MOFs have drawn a lot of attention over the past 20 years due to their promising applications for function engineering. MOFs are excellent candidates for the fabrication of sophisticated sensors due to their high porosity, abundance of structural characteristics, and diverse signal transductions. CMOFs have potential to be used in enantioselective applications. Yang and his coworkers fabricated gravimetric sensors by using CMOFs for enantioselective recognition of Cys enantiomer. Cys plays a vital role in biological processes, but its D-enantiomer has hazardous effect. So, UiO MOF has been modified and fabricated in the enantiomeric sensing device i.e. (UiO-tart@Au). In this device, D-enantiomer of Cys is captured by chiral MOF layer and mass of system increases by reaction of Au and Cys enantiomer. QCM has been used for gravimetric recognition of Cys D-enantiomer. DFT and SPE studies reveals that driving forces for enantioselectivity are Δ_r_H_m_ and H-bonding. This work paves path for development of devices for enantioselective application (Yang et al., 2021).

## 4 Conclusion

We have shown in the review the CMOFs are an emerging class of crystalline materials which has promising applications in various fields of life, MOFs are being used in different types of chromatographic techniques for separation, in catalysis, as capturing and luminescent material. Besides this MOFs can also be used as adsorbents and for degradation of materials MOFs structural diversity, porosity, thermal stability low density and high surface area have paved the way to use them efficiently in wide variety of applications. Different strategies have been used for synthesis of CMOFs, some of them are discussed in this review i.e., direct synthesis, indirect synthesis, and asymmetric induction. Among all these methods direct synthesis method is best for synthesis of CMOFs because by this method chirality of complete MOF can be achieved as well as greater chirality inductions makes MOFs suitable for different applications (Sharifzadeh et al., 2021). Chiral induction and spontaneous resolution methods have advantages of low cost and ease of synthesis but drawback of this method is formation of racemic conglomerates (Gong et al., 2022b). Novel CMOFs can be synthesized by using these synthetic routes.

### Challenges and future outlook

Various CMOFs have been developed and used for a range of applications. Herein, some of the synthesis strategies and applications of CMOFs were discussed. Although these are promising materials, there are a number of challenges that still exist which are needed to be resolved. For instance, the synthetic methods should be modified for balancing crystallographic symmetry and asymmetry, which forms due to chiral functional groups. Besides modifying synthetic methods, linkages can also be varied to overcome the issues. More efforts are needed to understand the mechanism for the transfer of chiral information and the nucleation process during crystallization because it is needed for more development in this field. CMOFs are successively used as a catalyst but one of the disadvantages of using MOFs as a catalyst is their instability. However, this problem can be solved by using secondary building units and different binding moieties. More research is needed to synthesize CMOFs, which are stable, reusable, and cost-effective. Besides CMOFs, covalent organic framework (COFs) also belongs to crystalline framework materials. COFs tunability, high porosity, structural predictability, and chemical diversity make them efficient materials for various applications (Altaf et al., 2021).

Besides all these properties, CMOFs also exhibit diverse abilities in them. In the upcoming years broad range of novel CMOFs will be reported having different asymmetric directions.

CMOFs contribute to the chiral world, so further research in this field will have a remarkable effect. The study on synthesizing CMOFs by varying synthetic strategies and linkages is a hot topic due to MOFs’ unique features and applications in every field of life.

## Glossary

ATH: Asymmetric transfer hydrogenation;
CSP: Chiral Stationary Phase;
Cys: Cysteine;
CMOFs: Chiral Metal Organic Framework;
CPL: Circularly Polarized Luminescence;
DFT: Density functional theory
DMF: N,N-Dimethyl-formamide
D/L-: cam
DL-: camphorates; ee Enantiomeric excess;
HPLC: High performance liquid chromatography;
GC: Gas chromatography;
OT: Open tubular;
CEC: Chiral electrochromatography;
CR: Congo red;
S-lac: S-lactate;
SPE: Solid Phase Extraction;
SURMOFs: Surface-mounted MOFs;
TBP: (1,2,4,5 tetra(pyridine-4-yl)benzene);
ILs: Ionic Liquids
QCM: Quartz crystal microbalance
MIDPPA: 4,4′-di (4-pyridine)-4″-imidazoletriphenylamine.
H_3_ChirBTB-1: 1,3,5-tri{4-[2-(4-isopropyl-2-oxooxazolidin-3-yl)] xcbenzoatebenzene.
H_3_ChirBTB-2: 1,3,5-tri {4-[2-(4-benzyl-2-oxooxazolidin- 3-yl)]benzoate}benzene
L_3_: 5-(9-anthracenyl) pyrimidine
L_4_: (S)-N-(pyridin-3-yl)-pyrrolidine-2-carboxamide)
L_5_: (S)-N-(pyridin-4-yl)-pyrrolidine-2-carboxamide)
1,2-pd: 1,2-propanediol.
H_6_L: hexa [4-(carboxyphenyl)oxamethyl]-3-oxapentane acid.
TPT: 4,6-tris(4-pyridyl)-1,3,5-triazine.
1L: { [ Zn_6_ (MIDP PA)_3_ (1, 2, 4-btc)_3_(NO_2_)_3_(H_2_O)_3_](H_2_O)_7_}*n*.
1R: (1,2,4- H_3_btc = 1,2,4-benzenetricarboxylic acid)
HL_2_: (S)-3-hydroxy-2-(pyridin-4-ylmethylamino)-propanoic acid.
Co.(d-Cam)1/2 (bdc)1/2 (tmdpy): (d-Cam = d-camphoric acid, bdc = 1,4-benzenedicarboxylic acid, tmdpy = 4,4′-trimethylenedipyridine)
{CaIICuII6L3(OH)2(H2O)}.nH2O: [1: L = (S,S)-alamox = [bis [(L)-alanine]oxalyldiamide] (n = 32); 2:L= (S,S)-valmox = [bis [(L)-valine]oxalyl diamide] (n = 13) and 3: L = (S,S)-leumox = [bis [(L)- leucine]oxalyl diamide] (n = 11)]
