# Supplementary figures and images for: Recent progress in the design, synthesis and applications of chiral metal-organic frameworks
Source: Front Chem. 2022 Oct 5;10:1014248. doi: 10.3389/fchem.2022.1014248 (PMC9581262; doi:10.3389/fchem.2022.1014248)

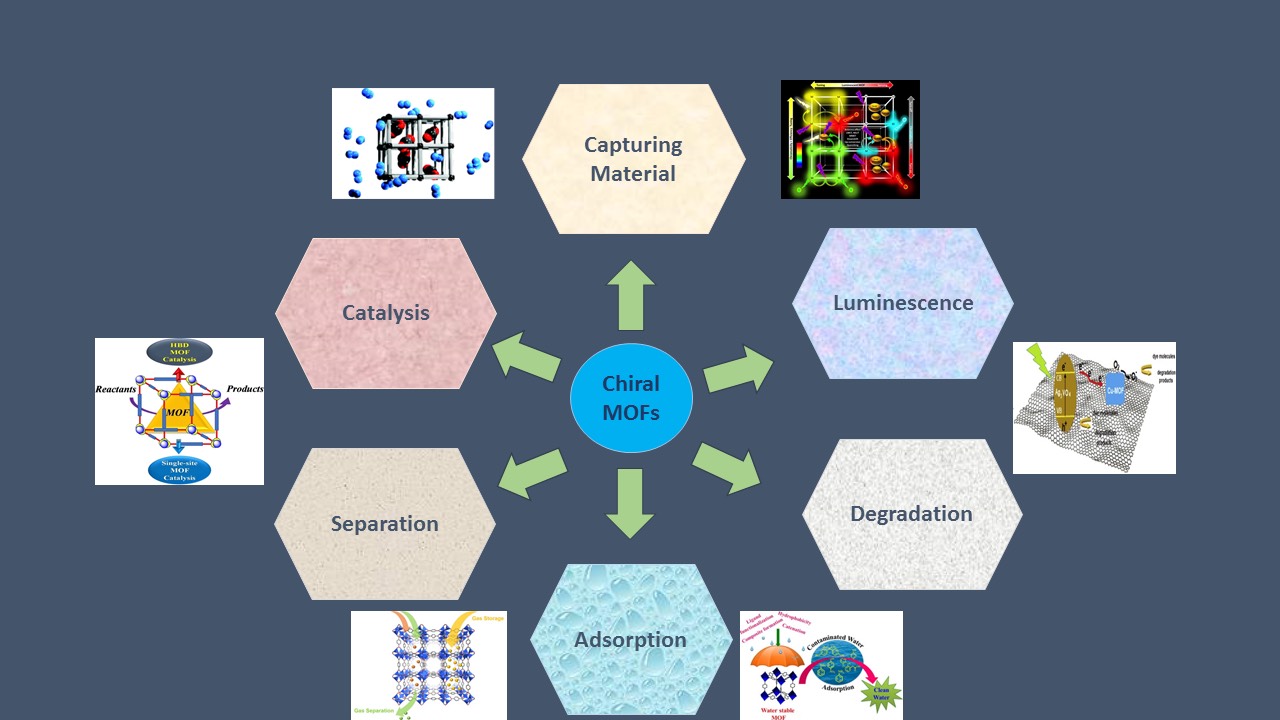

Supplement: Supplementary file 1 [file Image1.JPEG]
